# Combat-Related Post-traumatic Stress Disorder: A Case Report of Virtual Reality Graded Exposure Therapy With Physiological Monitoring in a U.S. Navy Officer and a U.S. Army Officer

**DOI:** 10.7759/cureus.19604

**Published:** 2021-11-15

**Authors:** Dennis P Wood, Michael J Roy, Brenda K Wiederhold, Mark D Wiederhold

**Affiliations:** 1 Virtual Reality and Psychology, Virtual Reality Medical Center, San Diego, USA; 2 Psychology, Private Practice, Marina, USA; 3 Department of Medicine, Uniformed Services University of the Health Sciences, Bethesda, USA; 4 Virtual Reality, Virtual Reality Medical Center, San Diego, USA

**Keywords:** outpatient treatment, biofeedback, post-traumatic stress disorder, combat, virtual reality graded exposure therapy, meditation, virtual reality exposure therapy, fmri, cerebral function, amygdala

## Abstract

The U.S. Department of Defense (DoD) and the Department of Veterans Affairs (DVA) seek to enhance the efficacy of treatments for warriors with post-traumatic stress disorder (PTSD) secondary to their combat deployments to Iraq and/or Afghanistan. Virtual Reality Graded Exposure Therapy (VR-GET) with arousal control has shown particular promise in reducing the symptom severity of PTSD in combat veterans. In this report, we describe the outcome of VR-GET for the treatment of combat-related PTSD in two combat veterans, neither of whom had received treatment for PTSD in the initial years after their return from combat duty.

## Introduction

Post-traumatic stress disorder (PTSD), also known as the “invisible wound” [1] or the “signature illness” [2] among Iraq and/or Afghanistan combat veterans, has an estimated prevalence rate of 10%-30% [1-8], with higher rates in those with multiple combat tours [3,7]. However, most combat veterans do not seek medical care [6] often because of stigma [1,2,6,8]. PTSD has also been associated with a double rate of hospitalization in active duty personnel from 2005 to 2012 and is the leading diagnosis in Veterans Administration (VA) Medical Centers [7].

Reports have suggested interventions and programs that could be implemented by military leaders or medical providers, as well as the VA, to reduce stigma and avoidance of treatment [1,3,6,8]. Aggressive early intervention strategies, therapies, and individualized treatment models have been recommended to improve the care of veterans with PTSD [1,3,6].

Exposure therapy, in which combat veterans “relive” the traumatic event in a graded and repeated process, has been successfully used to treat combat PTSD [9-11]. While imaginal exposure has demonstrable efficacy, avoidance of the reminders of the trauma is a defining feature of PTSD, and many combat veterans are unwilling or unable to effectively visualize their traumatic events [2,10,12], and the positive “response rate to exposure therapy is less than ideal” [11]. Virtual reality (VR) affords immersive, interactive, and realistic representation of the trauma, which can revolutionize exposure therapy for PTSD [2,9-14]. VR therapy overcomes many of the shortcomings of imaginal exposure by providing external visual and auditory stimuli for the combat veteran, eliminating the need for intense imaginal skills. Furthermore, VR therapy permits the combat veteran to interact with anxiety-inducing scenarios (e.g., combat, patrolling in a combat zone, driving in a Humvee, and providing medical care to a wounded Marine) in the safety and confidentiality of the therapy room [9,10].

Studies have demonstrated the efficacy of integrating physiological monitoring (e.g., heart rate, breath rate, skin conductance, and peripheral temperature) to further enhance VR therapy for PTSD [9,10,13,14]. We have previously shown that these physiological measures correlate with PTSD symptom severity [9,10,13,14]. We have also documented that physiological monitoring can enhance self-regulation, improve the degree of control over symptoms of hyperarousal, and augment therapists’ capacity to ascertain levels of arousal or discomfort during the conduct of VR exposure to optimize the pace and direction of therapy [9,10,13,14]. VR therapy, with physiological monitoring, has been characterized as Virtual Reality Graded Exposure Therapy (VR-GET).

To ascertain the effectiveness of VR-GET with Navy and Marine Corps personnel diagnosed with combat-related PTSD after deployment to Iraq or Afghanistan, the Office of Naval Research (ONR) funded research to evaluate the impact of VR-GET compared with treatment as usual (TAU) at the Naval Medical Center San Diego (NMCSD) and Naval Hospital Camp Pendleton (NHCP) [9,14]. The average PTSD and depression severity scores declined significantly for 12 pilot study participants who completed 10 VR-GET sessions [14], while in a randomized controlled trial, 7/10 receiving VR-GET, compared with only 1/9 receiving TAU, demonstrated a reduction in PTSD symptom severity of at least 30% [9].

A VR-GET case study reported a 20% reduction in the PTSD Checklist for Diagnostic and Statistical Manual of Mental Disorders, Fifth Edition (DSM-5) - Military Version (PCL-M) score in association with 10 VR-GET sessions for a Navy corpsman [13]. Another VR-GET case study documented that 20 VR-GET sessions conducted with a Seabee, who had comorbid PTSD and mild traumatic brain injury (mTBI) after three combat tours, reduced her PCL-M score by 65% [10].

A controlled trial that randomized military service members with combat PTSD to 12-20 sessions of VR-GET (n = 9) or prolonged imaginal exposure (PE; n = 10) reported modest but statistically significant improvement in PTSD symptom severity on the gold standard clinician-administered PTSD scale for those receiving VR-GET (80.4 at baseline to 64.5 posttreatment, p < 0.05), but not PE (72.7 at baseline to 75 posttreatment) [2,12]. Roy et al. also employed functional magnetic resonance imaging (fMRI), with the affective Stroop paradigm, to assess changes in regional brain activation levels before and after exposure therapy [2,12]. Some could not be scanned due to shrapnel or other issues, so they compared pre- and post-intervention scans for six PE and four VR-GET participants and found that the baseline hyperactivation in the amygdala and hippocampus resolved with exposure therapy, while inhibition in the anterior cingulate cortex significantly improved but did not entirely resolve. Since the post-intervention scans represented a second exposure to the affective Stroop for all participants, the improvement could in part represent a practice effect; thus, they also repeated fMRI scans at three-month intervals on 18 recently deployed combat veterans who did not have PTSD and found no difference between the first and the second scan in regional brain activation levels in response to the affective Stroop, providing additional credence that the difference observed in those with PTSD was due to the intervention, not practice or comfort with the affective Stroop [12].

Based on the preliminary evidence of the benefits described, in 2015, a male U.S. Navy officer, who had been diagnosed with combat-related PTSD and mTBI following his 2005 combat deployment to Iraq, was referred to one of us (DPW) for VR-GET by his primary care provider (PCP). In 2017, a U.S. Army officer, previously diagnosed with combat-related PTSD after his second combat tour in Iraq in 2004, received a similar referral. It should be noted that their VR-GET was provided in a private practice office rather than a military treatment facility.

## Case presentation

Methods

The diagnosis of probable combat-related PTSD was identified using the PTSD Checklist for Diagnostic and Statistical Manual of Mental Disorders, Fifth Edition (DSM-5) - Military Version (PCL-M) (Case 1) [15] or PTSD Checklist for DSM-5 (PCL-5) (Case 2) [16]. Clinical assessment, following the Diagnostic and Statistical Manual of Mental Disorders, Fifth Edition (DSM-5) [17] diagnostic guidelines, supplemented by the administration of the Patient Health Questionnaire-9 (PHQ-9) [18] and the Beck Anxiety Inventory (BAI) [19], identified comorbid adjustment disorder with mixed anxiety and depressed mood in Case 1 and major depressive disorder in Case 2 [18,19]. VR-GET was scheduled following the completion of this initial assessment in each case. Combat traumas and sentinel events were also identified.

Equipment

**Figure 1:**
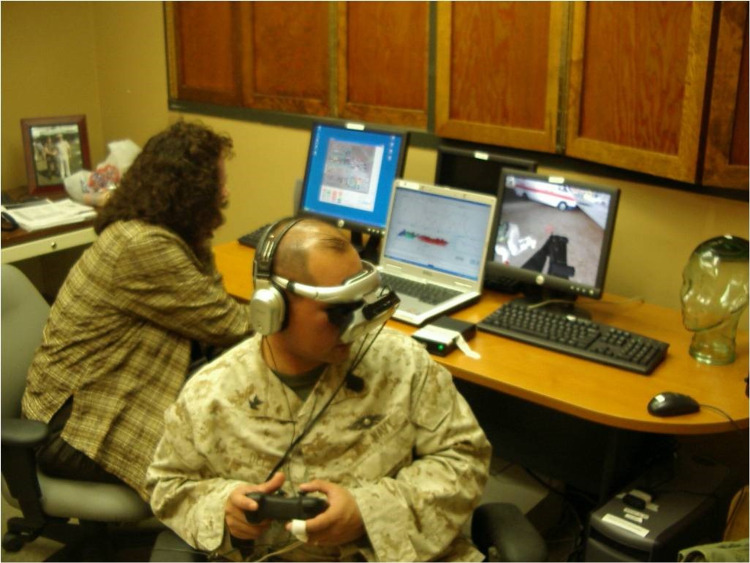
Three computer configurations for VR-GET with biofeedback being calculated on the laptop computer. Simulated combat veteran/non-patient is holding a handheld controller to “move” through the combat environment. A head-mounted display and headphones facilitate the immersion in the VR-GET simulated combat environment.

**Figure 2:**
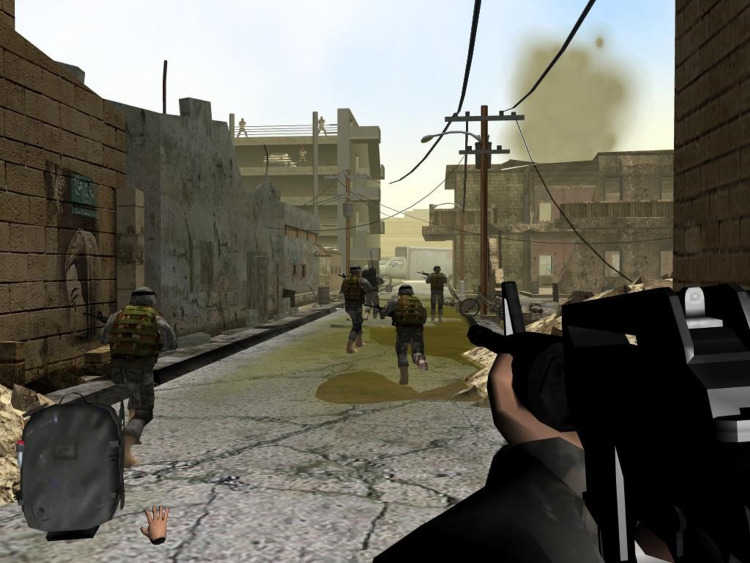
What the VR-GET combat veteran sees while immersed in the VR-GET combat environment titled “Fallujah.”

The VR worlds were designed and developed by the Virtual Reality Medical Center, San Diego, California (see Figures 1 and 2). The biofeedback system was developed by J & J Engineering, Poulsbo, Washington. The full VR-GET system was more fully described in a previous publication [10]. A recreation of a VR-GET session can also be viewed at https://www.youtube.com/watch?v=Y79sBIz2FvY.he [20].

Treatment

The VR-GET protocol utilized in each case encompassed 20 individual treatment sessions (90 minutes) that were conducted weekly or as often as possible if the service members’ schedules did not allow that. During VR-GET sessions 1 and 2, the patient is introduced to VR-GET and is also provided an introduction to meditation as a method for facilitating emotional, cognitive, and physical relaxation. Meditation training and practice are reinforced with a CD-based training program (Jon Cabot Zinn & Andrew Weil, Meditation for Optimum Health, Sounds True, Boulder, CO) to be practiced as homework.

During these first two VR-GET sessions, the patients are also asked to describe the sentinel events (i.e., the most traumatic events) during their combat deployments and their subsequent symptoms. The sentinel events were utilized as the exposure events within the VR-GET to foster arousal (e.g., increased heart rate and increased respiration). Meditation was utilized to foster decompression and a return to baseline levels of heart rate and respiration. The nature of PTSD and the corresponding goals of VR-GET to help achieve control over intrusive thoughts and feelings and learn to tolerate bothersome stimuli are also explained. In addition, the patients are taught the principles of attentional retraining (i.e., paying more attention to something enhances its impact, so paying attention to comfortable sensations at the moment, including while in the VR-GET combat environment, will enhance those thoughts and feelings, and should be favored over paying attention to thoughts and feelings that are uncomfortable).

Also, during the first two VR-GET sessions, each patient was physiologically monitored while they were exposed to a range of combat stimuli, for 20 minutes, in order to identify the types of combat-related stimuli and positive mental images that were most strongly associated with individual changes during the physiological arousal. For instance, heart rate and respiratory rate were evaluated during various VR combat scenarios, with the expectation that these physiological measures would increase with increased arousal. Conversely, when maximum arousal was achieved, instructions were given to “hold fire,” switch from a combat to a non-combat environment, and engage meditation and progressive relaxation efforts, with the expectation that measures of physiological arousal would decline. VR-GET then continues for an additional 18 sessions. A full description of the VR-GET has been previously published [10].

Case reports

Case 1

A 51-year-old male U.S. Navy captain with a history of PTSD and mild traumatic brain injury (mTBI) was referred by his Navy primary care provider (PCP) for VR-GET. He reported experiencing sleep disturbances, nightmares, excessive startle, flashbacks, and fear of dying in 2006 after a six-month deployment to Iraq but did not pursue a referral for treatment of his PTSD until 2015 because fellow officers suggested that his symptoms would improve with time. In addition to PTSD and mTBI, his past medical history was significant for hyperlipidemia and osteoarthritis, and he had been prescribed meloxicam, niacin, and atorvastatin. Zolpidem had previously been prescribed for sleep, but by the captain's fifth VR-GET session, zolpidem was replaced by escitalopram (10 mg daily) and trazodone (100 mg at bedtime), and sleep improved. Upon initial evaluation, comorbid adjustment disorder with mixed anxiety and depressed mood was also diagnosed. The captain had served for 25 years in the Navy, been married three times and divorced twice, and had three adult-aged children.

The captain’s sentinel events included but were not limited to the following: (1) “At approximately 0700 hours, in early February 2006, a suicide bomber detonated a ball bearing filled vest among a group of new Iraqi Army recruits. The detonation occurred approximately 1 km from Al Kasik’s Entry Control Point (ECP) in an area designated for the recruits to be prescreened to ensure that they were the confirmed recruits. A total of 65 recruits were killed or injured by the blast. Over 20 recruits died at the scene, and nine recruits died after medical evacuation as a result of their wounds. I was the scene leader and assisted with the collection of evidence. When I arrived at the scene, the smell of blood was still hanging in the air.” (2) “At approximately 1200 in mid-June 2006, I remembered dismounting and entering a town to interview a local official and inspect a local hospital and a fueling site. We were a sizable security group, but dismounting in the town was harrowing because we were targets. We spread out and walked in two long lines straddling the road through the town so that we all could not be taken out at once. The feeling was surreal. While holding perimeter security at the fueling site, my sector was approached by an unidentified intruder. I raised my rifle and yelled for him to stop in my best broken Arabic, but he refused to stop. Taking a bead on him, I called out to my interpreter to let the man know that he was going to be shot if he didn’t stop approaching. My interpreter was successful in keeping me from having to shoot the approaching man, and the intruder decided that it was best to go back the way he came.”

Following the completion of the initial 20 VR-GET sessions, utilizing the captain's sentinel events, the captain left on temporary duty assignments (TADs) for several months, and upon return, he requested additional VR-GET. Twelve more sessions were conducted over the next 10 months until he retired from the Navy. The PTSD Checklist for DSM-5 - Military Version (PCL-M) [15] was used to document PTSD symptom severity prior to the start of VR-GET and for reassessment after his completion of the first 20 VR-GET sessions, while the PTSD Checklist for DSM-5 (PCL-5) [16] was used to reassess symptom severity at the conclusion of the booster sessions, as the PCL-5 had come into use by that time.

**Figure 3:**
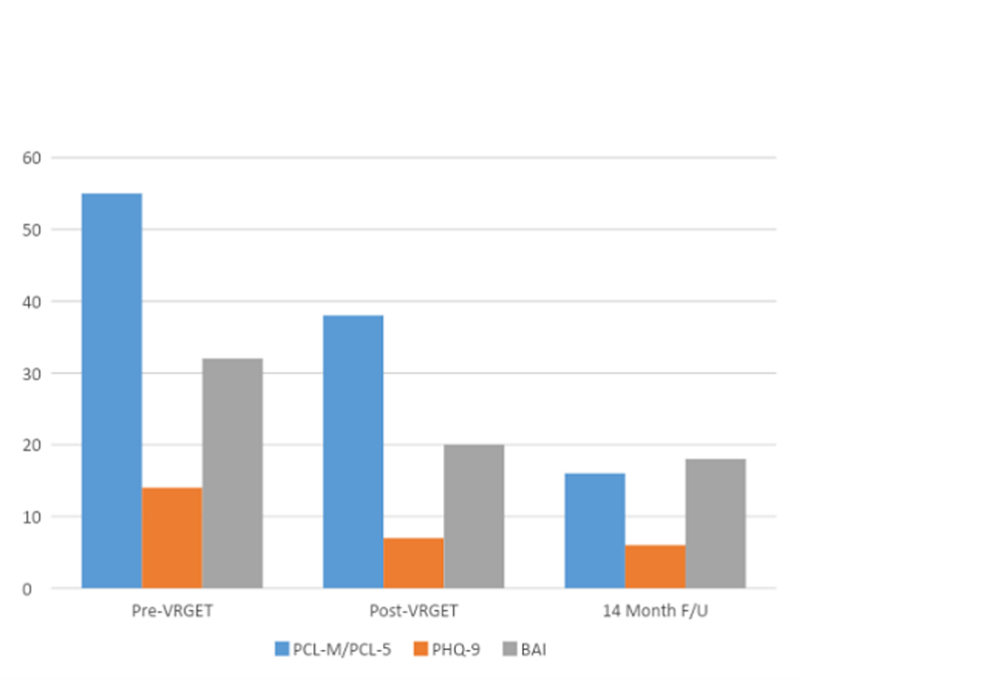
Results for PCL-M, PHQ-9, and BAI administered pre-VR-GET and post-VR-GET and results for PCL-5, PHQ-9, and BAI administered at 14 months follow-up.

After the first 20 VR-GET sessions, the captain's PTSD symptom severity had decreased measurably, and between the therapy and medication changes, his sleep had markedly improved. After the subsequent 12 VR-GET booster sessions, his PCL-5 score was 18, indicating resolution of the PTSD diagnosis (e.g., a PCL-5 cutoff score between 31 and 33 is indicative of probably PTSD) [16]. Likewise, his comorbid depression and anxiety markedly improved during the initial course of treatment, and this therapeutic gain was sustained over the period of booster sessions (see Figure 3).

Case 2

A 41-year-old male U.S. Army lieutenant colonel, diagnosed with PTSD and chronic insomnia by his Navy primary care provider, was referred for VR-GET. His past medical history was significant for obstructive sleep apnea, chronic sinusitis, gout, low back pain, and hypertension. He used continuous positive airway pressure (CPAP) nightly, and his medications included allopurinol, meloxicam, and nasal fluticasone. He had served for 18 years in the Army and was married with four children. He had four combat deployments to Southwest Asia, totaling 25 months, from 2003 to 2014. Although he was seen in the primary care setting for symptoms of PTSD after a 2004 deployment to Iraq, he was likewise reassured that his symptoms would get better with time, and thus, it was not until 2017 that he was referred for treatment. The clinical assessment confirmed the PTSD and, corroborated by the PHQ-9 [18], identified comorbid major depressive disorder. Low doses of nightly sertraline (50 mg every night at bedtime (qhs)) and quetiapine (50 mg qhs) were simultaneously initiated, and insomnia, mood, and anger began to improve. The lieutenant colonel initially completed 21 VR-GET sessions, utilizing his sentinel events, at which time he requested continued treatment, and another 18 VR-GET sessions were provided over the next 17 months, until his retirement from the Army.

The lieutenant colonel's sentinel events included but were not limited to the following. (1) “When just arriving at a large base in Iraq, with 20,000 U.S. personnel, we came under an indirect fire attack. These indirect fire attacks became a daily event, and we gradually became immune to these attacks and would even sleep through the alarms signaling the attacks. One more, while asleep, the attack alarm went off, and the rocket came in very low. This rocket passed directly over our tent and landed across the street with a loud explosion. The blast shook me out of my rack, and I ended up on the floor, and our tent was filled with dust, dirt, and debris. I began to look around our tent for one of my tentmates and began calling his name, and one of my other tentmates came into the tent only to tell me that my missing tentmate was already at work. Just then, four more rockets flew through our tent area and impacted close by, with one rocket impacting onto a tent and wounding the personnel inside.” (2) “Back in Iraq, two years later, our FOB was under attack from motor fire. I remember walking among my fellow officers in our barracks, telling everyone that it was going to be ok. I was lying and terrified on the inside but felt the need to maintain some ridiculous John Wayne persona.”

**Figure 4:**
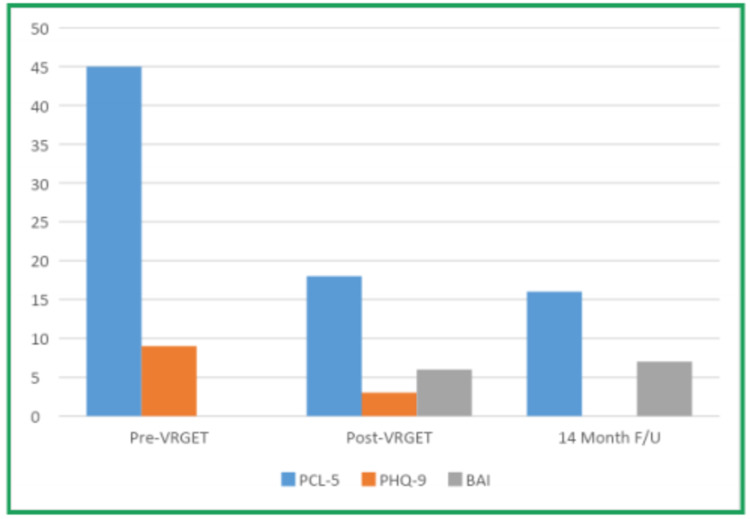
Results for PCL-5 administered pre- and post-VR-GET and at 14-month follow-up, results for PHQ-9 administered pre- and post-VR-GET, and results for BAI administered post-VR-GET and at 14-month follow-up (pretreatment BAI and 14-month follow-up PHQ-9 not available).

Following 21 VR-GET sessions, the lieutenant colonel’s PTSD symptom severity decreased measurably; between the therapy, CPAP, and medications, his sleep also largely returned to normal. These gains were sustained through the time of his retirement (see Figure 4).

## Discussion

Virtual Reality Graded Exposure Therapy (VR-GET) was well tolerated by these two senior military service members with notoriously difficult to treat combat-related PTSD, and it achieved marked reductions in PTSD symptom severity. The first had a modest reduction in symptoms by the end of the scheduled course of treatment but, with additional sessions over the subsequent 14 months, had resolution of PTSD, while the other achieved resolution by the end of the scheduled course and maintained that status in conjunction with continued, less frequent sessions. Both combat veterans reported significant engagement with the graded exposures afforded by VR, consistent with previous reports [9,10,13,14]. Specifically, they spontaneously and frequently commented on specific aspects of the VR, such as the road conditions, smells they recalled, and the dangerous appearance of the insurgents they encountered. Previously, another combat veteran had expressed, following the conclusion of her VR-GET, “I wished I had this training (e.g., meditation and exposure components) prior to my first combat deployment or between my combat deployments” [10]. Both combat veterans described in this report expressed a similar opinion regarding VR-GET at the conclusion of their treatment. The reduction in symptom severity and ultimate resolution of PTSD attest to the efficacy of the approach. These two case reports supplement the available literature regarding VR-GET [9,10,13,14], providing additional evidence for its utility, even when employed many years after the return from combat deployment and the onset of symptoms. The fact that the study was conducted in private practice, rather than a research or academic medicine environment, is also important, extending evidence of benefit to the setting in which most patients are seen.

However, there are several limitations to be acknowledged. The first case featured an initial assessment with the PCL-M, which has a score range of 17-85, and the subsequent assessment was completed with the PCL-5, which has a range from 0 to 80, so the scores cannot necessarily be compared directly, although the change that is evident is real, and it is clear that the initial score is consistent with a probable PTSD/PTS diagnosis (confirmed by clinical assessment), while the final score is consistent with resolution of PTSD/PTS. In addition, both patients had changes in their medications made in association with the initiation of VR-GET, so it cannot be definitively established how much of their improvement may be attributable to the medication changes as opposed to the VR-GET. There are also limitations to the generalizability of the results since a single clinical psychologist conducted the therapy using one VR-GET platform and one meditation training stimulus, so it cannot be inferred that success would occur with other therapists or other virtual reality treatment platforms. Finally, the impact of military retirement upon stress reduction for each of these senior officers might be significant, so the ultimate degree of improvement that can be attributed solely to VR-GET is uncertain. Additional studies are warranted to further corroborate these findings. Future studies should also assess whether pharmacologic augmentation can enhance the response to VR-GET, as well as whether there are demographic or other factors that can identify which patients are most likely to benefit from VR-GET.

## Conclusions

This two-case study documents that VR-GET can provide an attractive and effective private practice-based and individualized treatment for senior military personnel with combat-related PTSD.
